# Formation of Branched and Chained Alginate Microfibers Using Theta-Glass Capillaries

**DOI:** 10.3390/mi9060303

**Published:** 2018-06-17

**Authors:** Keigo Nishimura, Yuya Morimoto, Nobuhito Mori, Shoji Takeuchi

**Affiliations:** 1 Center for International Research on Integrative Biomedical Systems (CIBiS), Institute of Industrial Science (IIS), The University of Tokyo, 4-6-1 Komaba, Meguro-ku, Tokyo 153-8505, Japan; nishim@iis.u-tokyo.ac.jp (K.N.); y-morimo@iis.u-tokyo.ac.jp (Y.M.); mori1985@iis.u-tokyo.ac.jp (N.M.); 2 International Research Center for Neurointelligence (WPI-IRCN), The University of Tokyo Institutes for Advanced Study (UTIAS), The University of Tokyo, 4-6-1 Komaba, Meguro-ku, Tokyo 153-8505, Japan

**Keywords:** microfluidics, microfiber spinning, alginate hydrogel

## Abstract

This study proposes a microfluidic spinning method to form alginate microfibers with branched and chained structures by controlling two streams of a sodium alginate solution extruded from a theta-glass capillary (a double-compartmented glass capillary). The two streams have three flow regimes: (i) a combined flow regime (single-threaded stream), (ii) a separated flow regime (double-threaded stream), and (iii) a chained flow regime (stream of repeating single- and double-threaded streams). The flow rate of the sodium alginate solution and the tip diameter of the theta-glass capillary are the two parameters which decide the flow regime. By controlling the two parameters, we form branched (a Y-shaped structure composed of thick parent fiber and permanently divided two thin fibers) and chained (a repeating structure of single- and double-threaded fibers with constant frequency) alginate microfibers with various dimensions. Furthermore, we demonstrate the applicability of the alginate microfibers as sacrificial templates for the formation of chain-shaped microchannels with two inlets. Such microchannels could mimic the structure of blood vessels and are applicable for the research fields of fluidics including hemodynamics.

## 1. Introduction

Alginate microfibers have been attractive materials for biomedical applications such as cell and drug encapsulation [1,2,3], scaffolds for cell culture [4,5,6], and channel formation as sacrificial templates [7,8,9] because of their biocompatibility, biodegradability, and mechanical flexibility [1]. There have been two main methods to form the alginate microfibers: electrospinning and microfluidic spinning [10,11,12]. On one hand, electrospinning is suitable for forming high-resolution micro- and nano-scaled fibers, but the setup needs high voltages and there is a material limitation that alginate solutions cannot be independently electrospun without mixing them with other polymers such as chitosan [13,14]. On the other hand, microfluidic spinning, which involves a continuous extrusion of sodium alginate solution into a calcium chloride solution bath via microfluidic devices, has been widely used because of its easy setup and the adjustability of dimensions of microfibers by modulating flow conditions such as flow rates. Though resolutions of microfluidic spinning are lower than electrospinning, microfluidic spinning can form microfibers with various cross-sectional shapes. The alginate microfibers formed by microfluidic spinning have been shown to achieve not only uniform cross-sectional shapes when the extrusion of sodium alginate solution is constant (e.g., fiber [15,16,17], tube [18,19], and fiber with grooved surface [20,21]), but also cross-sectional shapes have been shown to change continuously when the extrusion is dynamically varied (e.g., coded fiber [20] and beads-on-a-string [22]). However, conventional microfluidic spinning methods can only generate a single-threaded microfiber, thus failing to form more complicated geometries such as in branched or chained microfibers.

**Figure 1:**
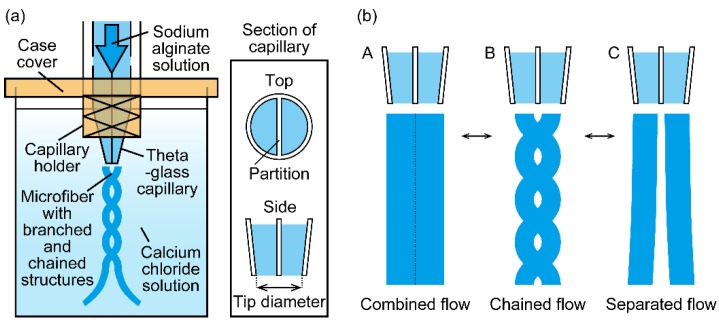
Schematic of the formation of alginate microfibers: (**a**) formation of alginate microfibers with branched structures using a theta-glass capillary; (**b**) conceptual illustrations of three flow regimes of two streams extruded from the theta-glass capillary (A: combined flow, B: chained flow, C: separated flow).

In this study, we present a microfluidic spinning method for the formation of branched/chained alginate microfibers by controlling the flow regime of a sodium alginate solution extruded from a theta-glass capillary (Figure 1a). This capillary has double compartments composed of a center partition and two parallel channels. In our experiment, we found that the two streams extruded from the capillary show three flow regimes: (i) a combined flow (single-threaded stream), (ii) a separated flow (double-threaded stream), and (iii) a chained flow (stream of repeating single- and double-threaded streams) based on the flow rate of the sodium alginate solution and the tip diameter of the theta-glass capillary (Figure 1b). Here, by controlling the flow regime, we try to fabricate alginate microfibers with branched/chained structures having various dimensions. We first reveal the relationship between the flow regimes and flow condition including the flow rate of the sodium alginate solution and the tip diameter of the theta-glass capillary, and then apply the method to form alginate microfibers with branched and chained structures. As a demonstration of the microfibers’ applicability, we form microchannels using the microfibers with branched structures as a sacrificial template.

## 2. Materials and Methods

### 2.1. Materials

To form alginate microfibers, we prepared a sodium alginate solution and 150-mM calcium chloride solution by dissolving sodium alginate powder (Junsei Chemical Co., Ltd., Tokyo, Japan) and calcium chloride powder (Kanto Chemical Co., Inc., Tokyo, Japan) in deionized water, respectively. To visualize the sodium alginate solution under a bright field or fluorescent microscopy, we added 5% (*v*/*v*) blue ink (PILOT Corporation, Tokyo, Japan) or 0.04% (*w*/*v*) fluorescent nanobeads (FluoSpheres™ carboxylate-modified microspheres, 0.2 µm, yellow-green fluorescent (505/515), 2% solids, Life Technologies Corp., Carlsbad, CA, USA) to the solution. As materials for microchannels, we used a polydimethylsiloxane (PDMS) elastomer and a curing agent (Silpot 184 W/C, Dow Corning Toray Co., Ltd., Tokyo, Japan). To form PDMS microchannels, we prepared a 500-mM sodium citrate solution as a chelating agent to wash out alginate microfibers by dissolving sodium citrate (Nacalai Tesque, Inc., Kyoto, Japan) in deionized water. To demonstrate a microfluidic operation in the PDMS microchannels, we prepared 5% (*v*/*v*) red ink (PILOT Corporation, Tokyo, Japan) and green ink (Pelikan Vertriebsgesellschaft mbH & Co. KG, Hannover, Germany) by diluting them with deionized water.

### 2.2. Device Fabrication

**Figure 2:**
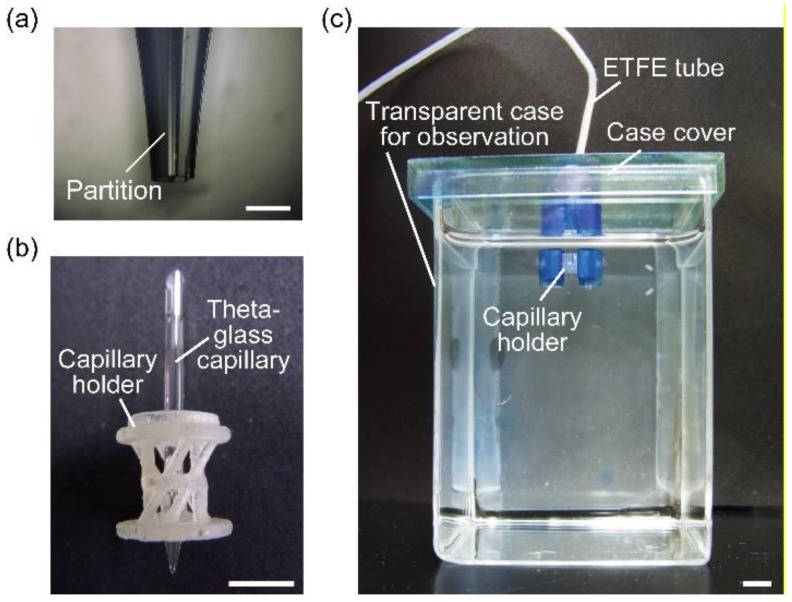
Experimental setups for alginate microfiber formation: (**a**) sharpened theta-glass capillary; (**b**) capillary holder assembled with the sharpened theta-glass capillary; (**c**) experimental setup for formation of alginate microfibers. Scale bars are: (**a**) 200 µm and (**b**,**c**) 5 mm.

The device used to form alginate microfibers was composed of a theta-glass capillary, capillary holder, and case cover. The tip of the theta-glass capillary (TST150-6, World Precision Instruments, Sarasota, FL, USA) was sharpened with a puller (PC-10, Narishige Co., Ltd., Tokyo, Japan). To adjust the tip diameters (tolerance: ±10 µm), we cut the sharpened tip using a microforge (MF-900, Narishige Co. Ltd., Tokyo, Japan) and grinded it using a microgrinder (EG-400, Narishige Co., Ltd., Tokyo, Japan) (Figure 2a). A capillary holder and case cover for immobilizing the theta-glass capillary were fabricated using a 3D printer (AGILISTA-3100, KEYENCE Corporation, Osaka, Japan).

### 2.3. Formation of Alginate Microfibers

A 5-mL syringe (Terumo Corp., Tokyo, Japan) containing a sodium alginate solution was connected to the theta-glass capillary with silicone tubes (AS ONE Corporation, Osaka, Japan) and ethylene tetrafluoroethylene (ETFE) tubes (VICI Precision Sampling, Inc., Baton Rouge, LA, USA). The theta-glass capillary was vertically set to the capillary holder and case cover and then placed on a clear case filled with a calcium chloride solution (Figure 2b,c). The theta-glass capillary tip was submerged under the calcium chloride solution, and the direction of the capillary outlet was approximately aligned to the direction of gravitational force. When the sodium alginate solution was extruded into the calcium chloride solution using a syringe pump (KDS 210, KD Scientific Inc., Holliston, MA, USA) under precise control of the flow rate, calcium-alginate hydrogel microfibers were formed by a crosslinking reaction of the sodium alginate solution with calcium ions. The formation of the microfibers was observed using a high-speed microscope (VW-9000, KEYENCE Corp., Osaka, Japan) and we classified the flow regimes according to the shapes of the alginate solution streams.

### 2.4. Fabrication of PDMS Microchannel Using Alginate Microfiber as Sacrificial Template

**Figure 3:**
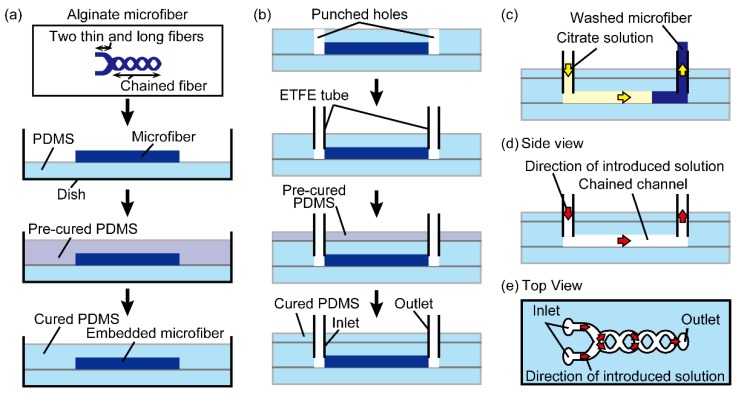
Process flow of the fabrication of chained PDMS microchannels. (**a**) Embedding of a microfiber with structure that two thin and long fibers are connected to a chained fiber into the PDMS substrate; (**b**) fabrication of inlets and an outlet for the microchannel; (**c**) removing the embedded microfiber; (**d**) side view of the formed microchannel; (**e**) top view of the formed microchannel.

To fabricate chained PDMS microchannels, we used a modified method of hydrogel molding techniques [8]. To briefly describe the process, we first prepared an alginate microfiber with a structure where two long and thin fibers are connected to a chained fiber by controlling the flow rates of the sodium alginate solution in order to switch the flow regimes (i.e., from separated to chained). A thin PDMS substrate was formed in a 60-mm cell culture dish (Corning Inc., Corning, NY, USA) by heating a PDMS-curing agent mixture at a ratio of 10 to 1 (*w*/*w*) on the dish bottom for 90 min at 75 °C. Next, the PDMS-curing agent mixture was poured onto the thin PDMS substrate with the alginate microfiber on its surface and cured by leaving it in a vacuum desiccator overnight at room temperature (Figure 3a). The PDMS structure with the alginate microfiber was removed from the dish and was punched with a biopsy punch (tip diameter 1.5 mm, Kai Industries Co., Ltd., Gifu, Japan) at the ends of the embedded microfiber to form one-side-open holes used as inlets and an outlet of a microchannel. To form inlets and an outlet without leakage, we inserted ETFE tubes in the holes and sealed the gap between the holes and tubes by forming a PDMS cover on the PDMS structure (Figure 3b). To form microchannels, we washed out the embedded microfiber by introducing 500-mM sodium citrate solution in the PDMS substrate through the inlets (Figure 3c). One of the fabricated PDMS microchannels (Figure 3d,e) was sliced into cross-sectional thin layers using disposable microtome knives (S35, FEATHER Safety Razor Co., Ltd., Osaka, Japan) in order to observe the cross sections. To demonstrate a microfluidic operation in the microchannel, 5% (*v*/*v*) red and green ink, which were loaded in 1-mL gastight syringes (Model 1001, Hamilton Co., Reno, NV, USA), were introduced using a syringe pump.

## 3. Results and Discussion

### 3.1. Characterization of Flow Regimes of Sodium Alginate Solution

**Figure 4:**
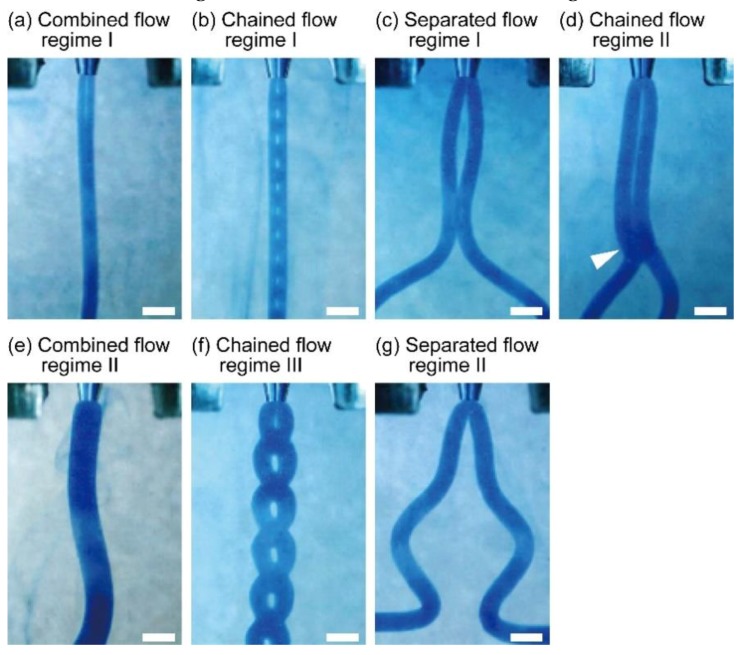
Flow regimes of a sodium alginate solution extruded into a calcium chloride solution at various flow rates through a theta-glass capillary: (**a**) combined flow regime I (0.01 mL/min); (**b**) chained flow regime I (0.03 mL/min); (**c**) separated flow regime I (3.0 mL/min); (**d**) chained flow regime II (4.1 mL/min). The arrowhead shows the combined part of the flow; (**e**) combined flow regime II (7.0 mL/min); (**f**) chained flow regime III (9.0 mL/min); (**g**) separated flow regime II (11 mL/min). Scale bars are 1 mm.

To investigate the variation of flow regimes of the sodium alginate solution extruded from the theta-glass capillary into the calcium chloride solution, we changed the flow rates of the sodium alginate solution to within a range of 0.01–15 mL/min. When we extruded 1.5% (*w*/*w*) sodium alginate solution into 150-mM calcium chloride solution through the theta-glass capillary with a 500-µm tip diameter, we observed that streams of the sodium alginate solution had various shapes depending on the flow rate (Figure 4, Movie S1). As we observed in the preliminary experiment, we confirmed that the streams can be classified into three flow regimes: combined flow regime (Figure 4a(I),e(II)), separated flow regime (Figure 4c(I),g(II)), and chained flow regime (Figure 4b(I),d(II),f(III)). In the combined flow regime occurring at 0.01 mL/min and 5.0–8.0 mL/min, a single-threaded stream was formed by integrating two streams at the capillary tip. In the separated flow regime occurring at 0.1–4.0 and 10–15 mL/min, a double-threaded stream was formed by retaining the two streams. By contrast, in the chained flow regime occurring at the narrow flow condition (0.02–0.05, 4.1, and 9.0 mL/min) between the combined and separated flow regimes, a chained stream was formed by repeated formations of single- and double-threaded streams. These results indicate that our method can generate streams of sodium alginate solution with various configurations. 

**Figure 5:**
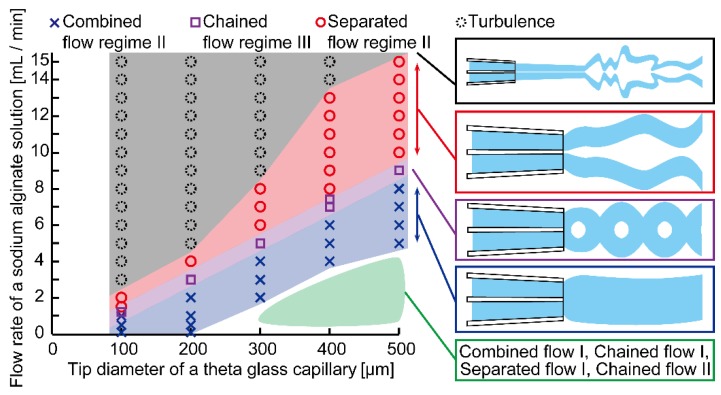
Experimental phases showing relationships between the flow regimes, flow rate of the sodium alginate solution, and tip diameter of the theta-glass capillary. Refer to Figure S1 for detailed data of the bottom-right regime.

Moreover, we investigated the relationships among the flow regimes, the flow rate of the sodium alginate solution, and the tip diameter of the theta-glass capillary and produced an experimental phase diagram (Figure 5 and Figure S1). Under the conditions of low flow rates (0.01–4 mL/min) and low tip diameters (100 and 200 µm), middle flow rates (2.0–8.0 mL/min) and a middle tip diameter (300 μm), and high flow rates (4.0–15 mL/min) and large tip diameters (400 and 500 μm), results showed that flow regimes were arranged in an orderly manner and that all three kinds of flow regimes appeared at every tip diameter. These flow regimes corresponded to combined flow regime II, chained flow regime III, and separated flow regime II observed in the aforementioned results. By contrast, under the conditions of low flow rates (0.01–4.0 mL/min) and large tip diameters (300–500 µm), we found that all three kinds of flow regimes, whose streams had smaller diameters, were also arranged in an orderly manner when tip diameters were 300 and 500 µm, but not in a perfectly orderly manner when the tip diameter was 400 µm. These flow regimes corresponded to combined flow regime I, chained flow regime I, separated flow regime I, and chained flow regime II (Figure S1). In addition, under the conditions of high flow rates (3–15 mL/min) and small tip diameters (100–400 µm), two streams aligned in parallel near the tip and deformed chaotically far away from the tip (Movie S2). These results indicate that the flow regime changed from separated flow regime II to turbulence because of increased flow speed. The turbulence is supposed to be caused by two factors: elastic turbulence [23] and inconstant fluidic properties of alginate during cross-linking. We also found that the flow rates required for the transition of flow regimes increased as the tip diameters of the theta-glass capillaries were lengthened. For example, when using theta-glass capillaries with a short tip diameter (100 µm), the transition of the flow regimes from combined flow regime II to separated flow regime II occurred at a low flow rate range (1.0–2.0 mL/min). By contrast, with a middle-length tip diameter (300 µm), the transition occurred at a middle flow rate range (4.0–6.0 mL/min), and with a long tip diameter (500 µm), the transition occurred at a high flow rate range (8.0–10 mL/min). These results indicate that the transition of the flow regimes is a robust phenomenon that occurs regardless of the lengths of the tip diameters of the theta-glass capillaries (i.e., at least within 100–500 µm).

### 3.2. Formation of Branched and Chained Alginate Microfibers

**Figure 6:**
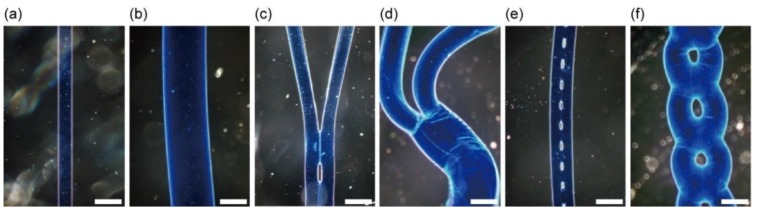
Alginate microfibers with various shapes: (**a**,**b**) single-threaded fibers formed in a combined flow regime (**a**) I and (**b**) II; (**c**,**d**) branched fibers formed in flow regimes changing from (**c**) combined flow regime I to separated flow regime I, and (**d**) combined flow regime II to separated flow regime II; (**e**,**f**) Chained fibers formed in chained flow regime (**e**) I and (**f**) II. Scale bars are 500 µm.

For the results of sodium alginate solution streams in various flow regimes extruded from the theta-glass capillary (500-µm diameter), we obtained alginate microfibers with various shapes, including single-threaded shapes (Figure 6a,b), branched structures (Figure 6c,d), and chained structures (Figure 6e,f). Formed alginate microfibers had different diameters based on the flow regimes. The single-threaded fiber formed in combined flow regime I (0.01 mL/min) (Figure 6a) had a shorter diameter than that of the microfiber formed in combined flow regime II (7.0 mL/min) (Figure 6b). The branched microfiber formed by switching from combined flow regime I (0.01 mL/min) to separated flow regime I (0.1 mL/min) (Figure 6c) also had shorter diameters than that of the microfiber formed by switching from combined flow regime II (7.0 mL/min) to separated flow regime II (11.0 mL/min) (Figure 6d). Similarly, the chained fiber formed in chained flow regime I (0.03 mL/min) (Figure 6e) had a shorter diameter than that of the microfiber formed in chained flow regime II (9.0 mL/min) (Figure 6f). These results indicate that the shapes of the alginate microfibers can be controlled based on the flow rates of the sodium alginate solution with varying diameters. To the best of our knowledge, ours is the first microfluidic method that can be used to form chained alginate microfibers.

**Figure 7:**
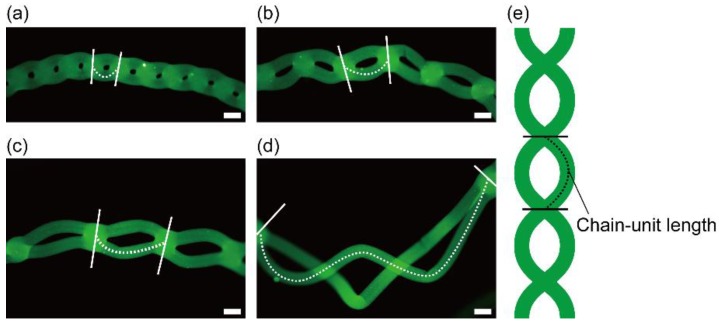
Fluorescent images of chained alginate microfibers with different chain-unit lengths. Microfibers were formed at flow rates of (**a**) 7.0 mL/min; (**b**) 7.5 mL/min; (**c**) 8.0 mL/min; and (**d**) 8.5 mL/min. The dotted line between two lines represents the chain-unit length; (**e**) Conceptual image of chain-unit lengths. Scale bars are 500 µm.

To evaluate the variability in the shape of chained alginate microfibers, we formed chained alginate microfibers by extruding 2.0% (*w*/*w*) sodium alginate solution containing fluorescent beads into a calcium chloride solution through the theta-glass capillary with a 400-µm tip diameter at different flow rates (7.0–8.5 mL/min) in the same flow regime (chained flow regime III) (Figure 7a–d). In this experiment, the range of flow rates generating chained flow regime III (7.0–8.5 mL/min) was different from that shown in Figure 5 because we used the sodium alginate solution with a different concentration (2.0% (*w*/*w*)) as well as a coloring agent (fluorescent nanobeads). As the flow rate of the sodium alginate solution increased, chain-unit lengths in chained microfibers, defined in Figure 7e, tended to increase. This result indicates that the lengths were variable according to the flow rate because the flow regime continuously changed from a combined flow regime (the length: 0) to a separated flow regime (the length: ∞).

### 3.3. Microchannel Formation with Chained Alginate Microfibers

**Figure 8:**
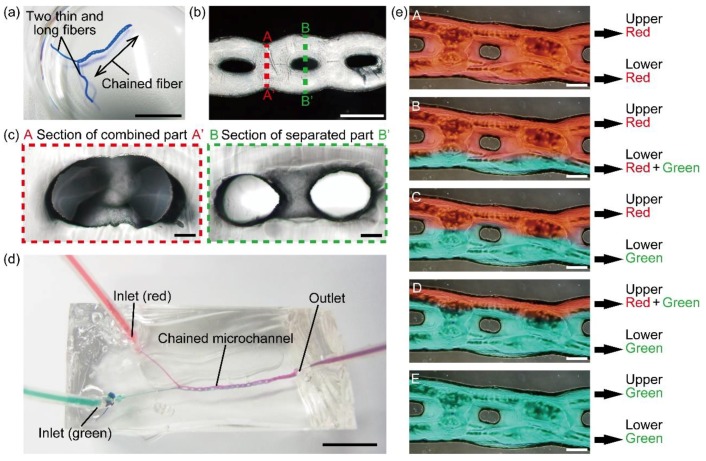
Chained PDMS microchannel fabricated with chained alginate microfibers. (**a**) Alginate microfiber with the structure that two thin and long fibers are connected to a chained fiber as a sacrificial template; (**b**) top view of the chained microchannel; (**c**) cross-sectional image of the microchannel shown in (**b**). Sections AA’ and BB’ are the combined and separated parts, respectively; (**d**) entire chained microchannel when infusing red and green solutions via different inlets; (**e**) flows of red and green solutions infused at A: 10 and 0, B: 8 and 2, C: 6.7 and 3.3, D: 5 and 5, E: 3.3 and 6.7, F: 2 and 8, and G: 0 and 10 mL/min, respectively. Scale bars are: (**a**,**d**) 1 cm; (**b**) 500 µm; (**c**,**e**) 100 µm.

To demonstrate the use of chained alginate microfibers as sacrificial templates, we formed a microfiber with a structure where two long and thin fibers are connected to a chained fiber by switching from a separated flow regime to chained flow regime (Figure 8a) and prepared a chained microchannel with two inlets by washing out the microfiber with a sodium citrate solution after embedding the microfiber in a PDMS structure (Figure 8b). The average diameters of the embedded alginate microfiber and the PDMS microchannel in each separated and combined region was 290 ± 18 and 295 ± 18 µm in separated regions, 681 ± 49 and 685 ± 26 µm in combined regions, respectively (Figure S2). The difference between the diameters of the alginate fiber and the PDMS microchannel was within 4% in both separated regions and combined regions. To check the shape of the microchannel, we observed the cross-section of the microchannel by slicing the PDMS structure into thin layers (approximately 200 µm thick). As a result, we confirmed that combined (single-hole channel) and separated (double-hole channel) parts were formed in the microchannel (Figure 8c). These results indicate that the alginate microfiber works as a sacrificial template that transfers its chained structure into the PDMS substrate. Compared to previous hydrogel template methods for forming branched microchannels which requires manual knotting [9] or arranging [8] of two threads of microfibers, our method can form a branched structure utilizing the microfluidic phenomenon, thereby improving the reproducibility of branched and chained shapes.

Finally, to confirm the function as a channel, we introduced two solutions colored with red and green ink into the microchannel through two inlets, respectively (Figure 8d). In this experiment, we varied the flow rate ratio of red and green solutions under five conditions: (i) a flow rate ratio of ∞ (10 mL red and 0 mL/min green solutions) (Figure 8eA), (ii) a flow rate ratio of 3 (7.5 mL red and 2.5 mL/min green solutions) (Figure 8eB), (iii) flow rate ratio of 1 (5 mL red and 5 mL/min green solutions) (Figure 8eC), (iv) flow rate ratio of 0.3 (2.5 mL red and 7.5 mL/min green solutions) (Figure 8eD), and (v) flow rate ratio of 0 (0 mL red and 10 mL/min green solutions) (Figure 8eE). As a result, laminar-like flows were formed, leading to five types of flows downstream: (i) both flows in the upper and lower channels were red (Figure 8eA), (ii) one upper flow was red and one lower flow was mixed (Figure 8eB), (iii) one upper flow was red and one lower flow was green (Figure 8eC), (iv) one upper flow was mixed and one lower flow was green (Figure 8eD), and (v) both flows in the upper and lower channels were green (Figure 8eE). The mixture ratio of red and green inks in this chained channel could be continuously variable as a result of controlling the flow rate ratio. These results indicate that a chained microchannel formed with our method is capable of liquid feeding and could be applied to the preparation of mixed solutions with various mixing ratios.

## 4. Conclusions

In this study, we developed a simple microfluidic method to form alginate microfibers with branched and chained structures using a theta-glass capillary. With our method, we could generate three flow regimes (combined, separated, and chained) by precisely controlling the flow rate of a sodium alginate solution and the tip diameter of the theta-glass capillary. By adjusting the flow regimes, we could change the cross-sections of flows to form alginate microfibers with various shapes such as single-threaded, branched, and chained microfibers. We were also able to change the microfiber dimensions such as the lengths, diameters, and chain-unit lengths in chained microfibers by regulating the flow rate and by changing the tip diameter of the theta-glass capillary. The advantages of our method are as follows: (i) devices can be easily prepared due to the commercial availability of theta-glass capillaries, (ii) our method offers a variety of complicated geometries and dimensions of microfibers, and (iii) branched and chained structures can be reproduced. Moreover, by switching between the different flow regimes, our method could form sacrificial microfibers with controlled positions of branched and re-combined structure; thus, the method has the potential to fabricate microchannels which mimic the structure of blood vessels featured with a multiple-branched structure or a specific chain-like vascular structure (e.g., vascular ring) and are therefore applicable for research fields of fluidics including hemodynamics.

## Supplementary Materials

The following is available online at http://www.mdpi.com/2072-666X/9/6/303/s1: Figure S1: Experimental phases showing the relationships among the flow rates of the sodium alginate solution, the tip diameters of the theta-glass capillaries, and the flow regime in the region with a low flow rate and large tip diameter; Figure S2: Diameters of the embedded microfiber and the PDMS microchannel; Movie S1: Flow regimes of the sodium alginate solution; Movie S2: Turbulent stream of the sodium alginate solution.
