# Supplementary material for: Formation of Branched and Chained Alginate Microfibers Using Theta-Glass Capillaries
Source: Micromachines (Basel). 2018 Jun 17;9(6):303. doi: 10.3390/mi9060303 (PMC6187737; doi:10.3390/mi9060303)
Supplement: Supplementary file 1 [file micromachines-09-00303-s001.zip › Supplementary Materials/K. Nishimura, et al. Figure S1 and S2.pdf]

Supplementary Materials: Formation of Branched and Chained Alginate Microfibers Using Theta-Glass Capillaries

Keigo Nishimura, Yuya Morimoto, Nobuhito Mori and Shoji Takeuchi *

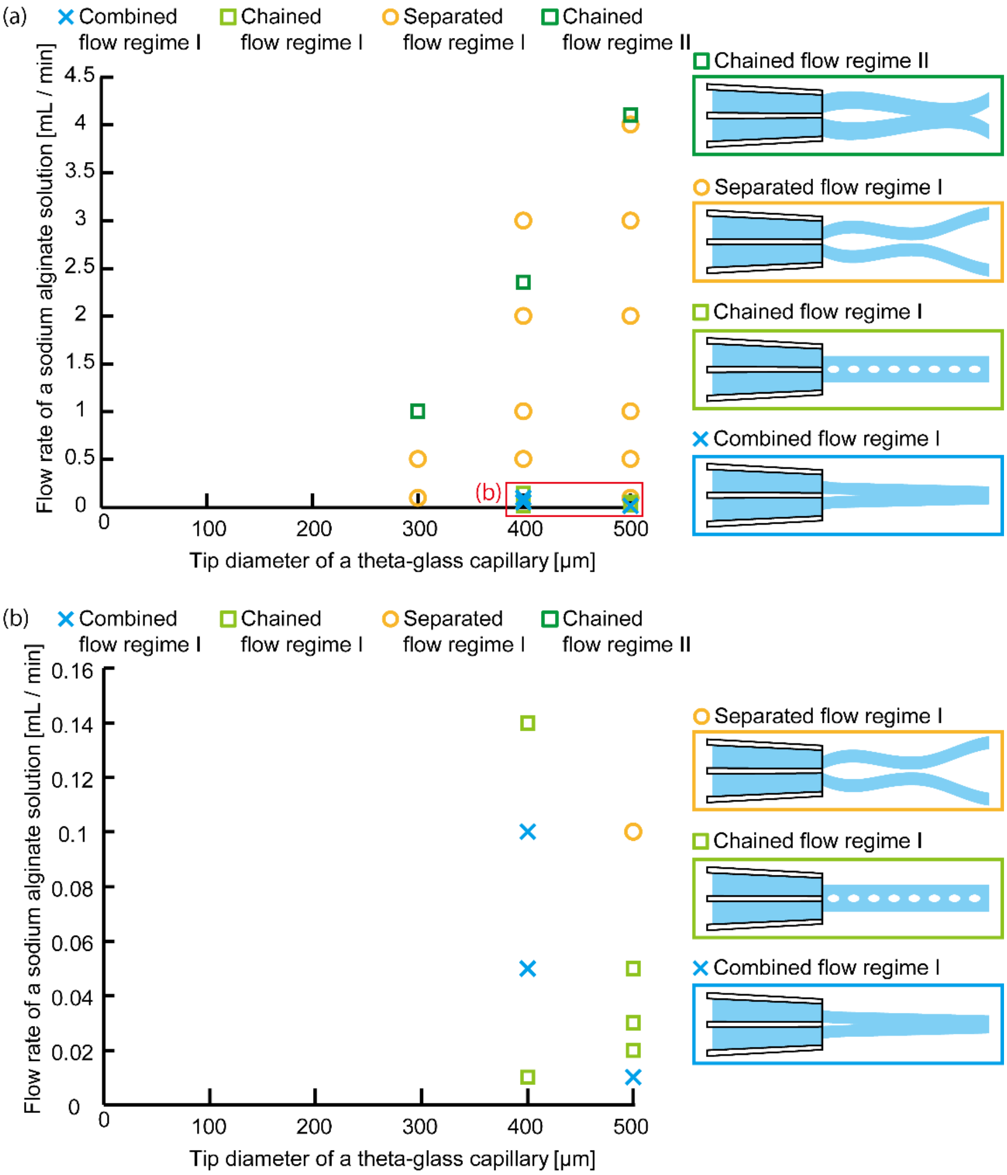

Figure S1. Experimental phases showing the relationships among the flow rates of the sodium alginate solution, the tip diameters of the theta-glass capillaries, and the flow regime in the region with a low flow rate and large tip diameter: (a) Experimental phases consisting of combined flow regime I, chained flow regime I, separated flow regime I, and chained flow regime II; (b) Enlarged view of red lined area in (a).

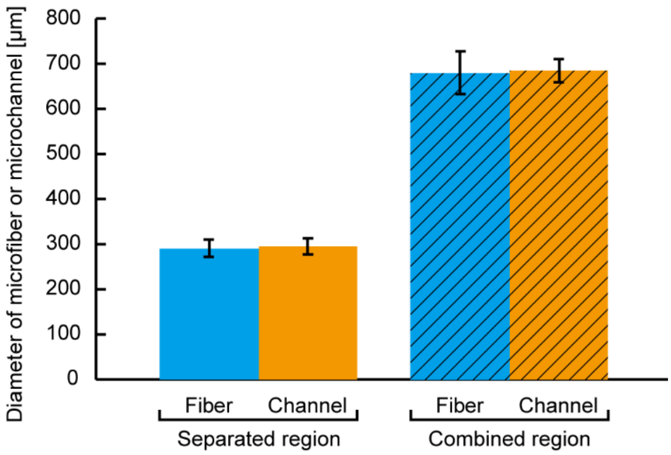

Figure S2. Diameters of the embedded microfiber and the PDMS microchannel; n=3; Error bars: S.D.
